# Obituary

**Published:** 1909-11-13

**Authors:** 


### OBITUARY.

We have to record the death of Dr. James Edward Scott, of Dublin, on November 7. Dr. Scott took his degree of M.B. at Dublin in 1847, having become M.R.C.S. of England the previous year. The Dublin M.D. was conferred on him in 1854. Dr. Scott was also deputy surgeon-general of the A.M.S. and late Surgeon-Major 4th Battalion Rifle Brigade. He was 84 when he died.

The death is announced, at Bournemouth, of Dr. John Waggett, M.D., F.R.C.S. He took his degree of M.D. at Edinburgh in 1842, and became a Fellow of the Royal College of Surgeons of England in 1860. He studied at University College, London, and was afterwards a member both of the British Medical Association and also a Fellow of the Medical Society of London. He made his home at Bournemouth, and was in his ninety-first year at the time of his death.

We have to record the death of Mr. Henry Hugh Clutton, senior surgeon at St. Thomas's Hospital, on November 9. Mr. Clutton studied at St. Thomas's Hospital and took his M.R.C.S. in 1875, becoming a Fellow of the College the following year. He took the degree of M.D. in 1896. He was a member of the Council of the Royal College of Surgeons, and representative of the College on the Senate of the University of London, and a member of the Visiting Committee of King Edward's Hospital Fund. He was also Examiner in Surgery at Cambridge and surgeon to the Victoria Hospital for Children. He wrote several articles in various text-books on surgery, and contributed to Societies' Transactions.

